# A phase 1b, open-label study of trebananib plus bevacizumab or motesanib in patients with solid tumours

**DOI:** 10.18632/oncotarget.2568

**Published:** 2014-10-23

**Authors:** David S. Hong, Razelle Kurzrock, Marilyn Mulay, Erik Rasmussen, Benjamin M. Wu, Michael B. Bass, Zhandong D. Zhong, Greg Friberg, Lee S. Rosen

**Affiliations:** ^1^ Department of Investigational Cancer Therapeutics, Division of Cancer Medicine, University of Texas MD Anderson Cancer Center, 1515 Holcombe Blvd, Unit 455, Houston, TX 77230–1402, USA; ^2^ Center for Personalized Cancer Therapy and CTO, Division of Hematology and Oncology, UC San Diego Moores Cancer Center, 3855 Health Sciences Drive, MC #0658, La Jolla, CA 92093–0658, USA; ^3^ Mulay Educational and Clinical Consulting Associates, 12412 Texas Ave., Suite 206, Los Angeles, CA 90025, USA; ^4^ Department of Biostatistics, Amgen Inc., One Amgen Center Drive, Thousand Oaks, CA 91320, USA; ^5^ Department of Pharmacokinetics and Drug Metabolism, Amgen Inc., One Amgen Center Drive, Thousand Oaks, CA 91320, USA; ^6^ Department of Molecular Sciences and Computational Biology, Amgen Inc., One Amgen Center Drive, Thousand Oaks, CA 91320, USA; ^7^ Department of Clinical Immunology and Biological Sample Management, Amgen Inc., One Amgen Center Drive, Thousand Oaks, CA 91320, USA; ^8^ Department of Early Development, Amgen Inc., One Amgen Center Drive, Thousand Oaks, CA 91320, USA; ^9^ Department of Medicine, Division of Hematology and Oncology, UCLA, Santa Monica, CA 90404, USA

**Keywords:** angiogenesis, angiopoietins, Tie2 receptor, vascular endothelial growth factor, angiogenic inhibitors

## Abstract

**Background:**

To examine the angiopoietin pathway inhibitor trebananib IV plus the anti-VEGF agents bevacizumab or motesanib in advanced solid tumours.

**Methods:**

In this open-label phase 1b study, patients received IV trebananib 3 mg kg^−1^ QW plus bevacizumab 15 mg kg^−1^ Q3W (cohort 1) or motesanib orally 75 mg (cohort 2); or trebananib 10 mg kg^−1^ plus bevacizumab 15 mg kg^−1^ (cohort 3) or motesanib 125 mg (cohort 4). If <33% of patients had dose-limiting toxicities (DLTs), dose escalation occurred. Endpoints were treatment–related adverse events (AEs) incidence and pharmacokinetics (primary); anti-trebananib antibodies, biomarkers, and tumour response (secondary).

**Results:**

Thirty-six patients received ≥1 dose of trebananib (cohorts 1, 2, 3, 4; *n* = 6, 8, 19, 3). DLT of G3 intestinal perforation and G3 tumor haemorrhage occurred in cohorts 2 and 3, respectively (both *n* = 1). Across both trebananib plus bevacizumab cohorts, the most common AEs included fatigue (*n* = 8), diarrhoea (*n =*4), constipation (*n* = 3), nausea (*n* = 3), and epistaxis (*n =* 3). Three patients across those cohorts had grade ≥3 AEs. Across the trebananib plus motesanib cohorts, the most common AEs included hypertension (*n =* 4), diarrhoea (*n* = 4), nausea (*n =* 3), fatigue (*n* = 3), vomiting (*n* = 2), and decreased appetite (*n* = 2). Two patients had grade ≥3 AEs. Trebananib did not markedly affect motesanib pharmacokinetics. Across the trebananib plus bevacizumab cohorts, two patients had a partial response; 11 patients had stable disease lasting >6 months. Across the trebananib plus motesanib cohorts, one patient had a partial response; five patients had stable disease lasting >6 months.

**Conclusion:**

Trebananib IV 3 mg kg^−1^ or 10 mg kg^−1^ plus bevacizumab or motesanib in advanced solid tumours may be associated with less severe toxicities relative to those emerging when combining two anti-VEGF agents.

## INTRODUCTION

Angiogenesis, the process of formation of new blood vessels from the pre-existing vasculature, is critically important in tumour development and metastasis [1]. Relatively recent approaches in advancing antiangiogenic therapies for cancer have involved simultaneous inhibition of multiple angiogenic targets in an effort to achieve superior efficacy relative to inhibition of single targets. To date, these attempts have been limited to the combination of anti-VEGF pathway-targeted therapies. While there appears to be some suggestion of enhanced efficacy, increased toxicities were also observed, likely as a result of additive, synergistic, or antagonistic stimulation of the VEGF pathway [2]. Moreover, combination treatments targeting the same pathway carry the risk of initiating compensatory escape mechanisms [3–5]. Preclinical research suggests that the combination of an angiopoietin inhibitor and an anti–VEGF antibody may enhance antitumor activity [6]. It is unknown, however, whether such combination therapies would provide an improved risk/benefit ratio in the clinical setting.

The angiopoietin pathway is a key regulator of angiogenesis [7, 8]. It involves the receptor tyrosine kinase, Tie2, which is expressed in a limited number of cell types, including the vascular endothelium [9]. Tie2 binds the ligands Ang1, Ang2, and Ang4, with Ang1 and Ang2 being well characterised. While Ang1 appears to contribute to vessel maturation and stabilisation [10], Ang2 plays a crucial role in vessel destabilisation during vascular remodelling [11] and new vessel sprouting [9]. Thus, both represent important and complementary determinants of angiogenesis. Elevated Ang2 expression has been found in tumour vasculature across various cancer types and has been associated with disease progression [11–13] and worse prognosis [14].

Trebananib, an investigational, intravenously administered peptide-Fc fusion protein (“peptibody”), inhibits tumour angiogenesis through dual inhibition of Ang1 and Ang2, thereby neutralising their interaction with the Tie2 receptor. Animal models have demonstrated tumour inhibition when trebananib is administered systematically to tumour-bearing mice [11]. Importantly, the antitumor effect has been shown to be greater with combined inhibition of Ang1 and Ang2 compared with blocking of either ligand in isolation [15]. Results from a first-in-human dose escalation study in patients with advanced solid tumours showed that trebananib monotherapy had antitumor activity and was tolerated at doses up to 30 mg kg^−1^ once weekly (QW) with a toxicity profile that was distinct from other angiogenesis inhibitors, including VEGF pathway inhibitors [16]. The most common treatment-related toxicities during trebananib monotherapy included peripheral oedema and fatigue; no bleeding or thromboembolic events occurred. A randomised phase 2 study in patients with recurrent ovarian cancer suggested that treatment with trebananib at 3 mg kg^−1^ or 10 mg kg^−1^ QW plus paclitaxel QW may result in improved progression-free survival (PFS) relative to placebo plus paclitaxel QW, particularly at the higher dose [17]. Again, the toxicity profile was distinct and generally manageable.

The current study's objectives were to assess the tolerability, pharmacokinetic (PK) and biomarker profiles, and tumour response to trebananib in combination with two VEGF pathway inhibitors, the humanised recombinant monoclonal antibody bevacizumab or small molecule antagonist motesanib, in adult patients with advanced solid tumours. Bevacizumab inhibits VEGF with demonstrated antitumor activity in combination with multiple chemotherapies in several cancers such as colorectal and non–small cell lung cancer [18, 19]. Motesanib blocks the VEGF receptors (VEGFRs)-1, -2, and -3, platelet-derived growth factor receptor (PDGFR), and c-Kit [20]. In previous studies, motesanib treatment reduced tumour burden when administered as monotherapy or in combination with chemotherapy [21–23].

## RESULTS

### Patients

A total of 38 patients were enrolled between December 2005 and February 2007: cohort 1 (trebananib 3 mg kg^−1^ plus bevacizumab 15 mg kg^−1^), *n =* 6); cohort 2 (trebananib 3 mg kg^−1^ plus motesanib 75 mg), *n =* 9; cohort 3 (trebananib 10 mg kg^−1^ plus bevacizumab 15 mg kg^−1^) *n =* 20; and cohort 4 (trebananib 3 mg kg^−1^ plus motesanib 125 mg) *n =* 3. One patient each in cohorts 2 and 3 did not receive trebananib treatment and, therefore, was excluded from all subsequent analyses. Patients discontinued the study for the following reasons: disease progression (cohorts 1, 2, 3, 4; *n =* 2, 3, 13, 2), death (*n =* 1, 3, 2, 0), withdrawal of consent (*n =* 0, 1, 1, 0), adverse events (*n =* 1, 0, 0, 0), and other (*n =* 2, 2, 4, 1).

Across cohorts 1 through 4, patients received a median of 18.5, 9.5, 15.0, and 11.0 weekly doses of trebananib, respectively. Cohorts 1 and 3 were administered a median number of 7.0 and 6.0 doses of bevacizumab, respectively. Cohorts 2 and 4 were given a median number of 66.5 and 87.0 doses of daily motesanib, respectively. Demographic and baseline characteristics are depicted in Table 1. Patients' duration of study participation is provided in Figure 1 of the Supplemental Data section.

**Table 1:** Demographic and disease characteristics

	Cohort 1	Cohort 3	Cohort 2	Cohort 4	Cohorts 1–4
	Trebananib 3 mg kg^−1^ + bevacizumab 15 mg kg^−1^ (*n* = 6)	Trebananib 10 mg kg^−1^ + bevacizumab 15 mg kg^−1^ (*n* = 19)	Trebananib 3 mg kg^−1^ + motesanib 75 mg (*n* = 8)	Trebananib 3 mg kg^−1^ + motesanib 125 mg (*n* = 3)	Trebananib + bevacizumab or motesanib (*n* = 36)
**Sex,** *n* **(%)**					
Female	2 (33)	13 (68)	4 (50)	2 (67)	21 (58)
Male	4 (67)	6 (32)	4 (50)	1 (33)	15 (42)
**Race/ethnicity,** *n* **(%)**					
Caucasian	5 (83)	14 (74)	8 (100)	3 (100)	30 (83)
African American	0 (0)	3 (16)	0 (0)	0 (0)	3 (8)
Hispanic	1 (17)	1 (5)	0 (0)	0 (0)	2 (6)
Asian	0 (0)	1 (5)	0 (0)	0 (0)	1 (3)
**Age, median (range), years**	61.5 (33, 73)	56 (38, 73)	53.5 (37, 80)	55 (51, 67)	56 (33, 80)
**ECOG score,** *n* **(%)**					
0	2 (33)	7 (37)	3 (38)	2 (67)	14 (39)
1	4 (67)	11 (58)	5 (63)	1 (33)	21 (58)
2	0 (0)	1 (5)	0 (0)	0 (0)	1 (3)
**Primary tumour type,** *n* **(%)**					
Breast cancer	1 (17)	8 (42)	0 (0)	0 (0)	9 (25)
Prostate cancer	1 (17)	3 (16)	0 (0)	0 (0)	4 (11)
Uterine cancer	1 (17)	2 (11)	1 (13)	0 (0)	4 (11)
Ovarian cancer	0 (0)	0 (0)	2 (25)	2 (67)	4 (11)
Head and neck squamous cell cancer	1 (17)	1 (5)	0 (0)	0 (0)	2 (6)
Oesophageal cancer	0 (0)	0 (0)	2 (25)	0 (0)	2 (6)
Pancreatic cancer	1 (17)	1 (5)	0 (0)	0 (0)	2 (6)
Small cell lung cancer	1 (17)	0 (0)	1 (13)	0 (0)	2 (6)
Testicular cancer	0 (0)	0 (0)	1 (13)	0 (0)	1 (3)
Ureteral cancer	0 (0)	0 (0)	1 (13)	0 (0)	1 (3)
Melanoma	0 (0)	1 (5)	0 (0)	0 (0)	1 (3)
Non-small cell lung cancer	0 (0)	1 (5)	0 (0)	0 (0)	1 (3)
Soft-tissue sarcoma	0 (0)	1 (5)	0 (0)	0 (0)	1 (3)
Other	0 (0)	1 (5)	0 (0)	1 (33)	2 (6)
**Lines of prior chemotherapy,** *n* **(%)**					
0	0 (0)	1 (5)	0 (0)	0 (0)	1 (3)
1	0 (0)	2 (11)	1 (13)	0 (0)	3 (8)
2	2 (33)	2 (11)	2 (25)	1 (33)	7 (19)
≥3	4 (67)	14 (74)	5 (63)	2 (67)	25 (69)
**Any prior radiotherapy,** *n* **(%)**					
Yes	5 (83)	12 (63)	2 (25)	0 (0)	19 (53)
No	1 (17)	7 (37)	6 (75)	3 (100)	17 (47)
**Any prior cancer-related surgery,** *n* **(%)**					
Yes	5 (83)	17 (89)	7 (88)	3 (100)	32 (89)
No	1 (17)	2 (11)	1 (13)	0 (0)	4 (11)

### Tolerability

No DLTs occurred in cohort 1 (trebananib 3 mg kg^−1^ plus bevacizumab 15 mg kg^−1^). Nonetheless, after one of the initial three enrolled patients died from arterial haemorrhage at week 10, the decision was made to expand the current cohort to six patients before initiating enrolment into the higher dose cohort (cohort 3). One patient in cohort 2 (trebananib 3 mg kg^−1^ plus motesanib 75 mg) had a DLT of intestinal perforation; therefore, cohort 2 was also expanded to include six patients. Two patients in cohort 2 did not receive trebananib and motesanib simultaneously during the first study month on days when both therapies were administered; thus, two new patients were added for a total of eight patients. In cohort 3 (trebananib 10 mg kg^−1^ plus bevacizumab 15 mg kg^−1^), one patient had a DLT of tumour haemorrhage. No DLTs occurred in cohort 4 (trebananib 3 mg kg^−1^ plus motesanib 125 mg). Five patients in cohort 3 received fewer than three doses of trebananib as the result of a DLT (*n* = 1) and disease progression (*n* = 4); thus, the cohort was expanded to include an additional three patients. Investigators opted to add 10 more patients to cohort 3 for a final cohort enrolment of 19 patients.

This report presents treatment-related adverse events that were considered to be possibly related to any of the administered study agents per the clinical investigator's assessment. No cohort-specific trends in the incidence of treatment-related adverse events across treatment groups were noted. Across the dose cohorts of trebananib plus bevacizumab (cohorts 1 and 3, *n =* 25), the most common treatment-related adverse events included fatigue, diarrhoea, constipation, nausea, and epistaxis (Table 2). Three patients (12%) had grade ≥3 treatment-related adverse events, including arterial haemorrhage (grade 5; *n* = 1) in a patient with squamous cell head and neck cancer in cohort 1, tumour haemorrhage (grade 5; *n* = 1) in a patient with squamous cell head and neck cancer, and fatigue (grade 3; *n* = 1) in a patient with breast cancer in cohort 3. No grade 4 treatment-related adverse events occurred in cohorts 1 or 3. In addition to the patients with arterial haemorrhage and tumour haemorrhage in cohorts 1 and 3, respectively, two patients in cohort 3 died from disease progression (*n =* 1) and respiratory failure (*n =* 1). Those two deaths were not considered to be related to the study treatment.

**Table 2:** Patient incidence of treatment–related adverse events in the trebananib plus bevacizumab cohorts

	Cohort 1		Cohort 3		Cohorts 1–3	
	Trebananib 3 mg kg^−1^ + bevacizumab 15 mg kg^−1^ (*n* = 6)		Trebananib 10 mg kg^−1^ + bevacizumab 15 mg kg^−1^ (*n* = 19)		Trebananib + bevacizumab (*n* = 25)	
Patients with any treatment-related adverse event^a^, *n* (%)	5 (83)		10 (53)		15 (60)	
Grade 3	0 (0)		1 (5)		1 (4)	
Grade 4	0 (0)		0 (0)		0 (0)	
Grade 5	1 (17)^b^		1 (5)^c^		2 (8)	
**Treatment-related adverse events occurring in one or more treatment arms,** *n* **(%)**	**All grades**	**Grade ≥ 3**	**All grades**	**Grade ≥ 3**	**All grades**	**Grade ≥ 3**
Fatigue	3 (50)	0 (0)	5 (26)	1 (5)	8 (32)	1 (4)
Diarrhoea	1 (17)	0 (0)	3 (16)	0 (0)	4 (16)	0 (0)
Constipation	0 (0)	0 (0)	3 (16)	0 (0)	3 (12)	0 (0)
Nausea	1 (17)	0 (0)	2 (11)	0 (0)	3 (12)	0 (0)
Epistaxis	2 (33)	0 (0)	1 (5)	0 (0)	3 (12)	0 (0)
Chest discomfort	1 (17)	0 (0)	1 (5)	0 (0)	2 (8)	0 (0)
Arthralgia	1 (17)	0 (0)	1 (5)	0 (0)	2 (8)	0 (0)
Cough	0 (0)	0 (0)	2 (11)	0 (0)	2 (8)	0 (0)
Hypertension	0 (0)	0 (0)	2 (11)	0 (0)	2 (8)	0 (0)
Arterial haemorrhage	1 (17)	1 (17)	0 (0)	0 (0)	1 (4)	1 (4)
Tumour haemorrhage	0 (0)	0 (0)	1 (5)	1 (5)	1 (4)	1 (4)
Myopia	0 (0)	0 (0)	1 (5)	0 (0)	1 (4)	0 (0)
Lower abdominal pain	0 (0)	0 (0)	1 (5)	0 (0)	1 (4)	0 (0)
Vomiting	0 (0)	0 (0)	1 (5)	0 (0)	1 (4)	0 (0)
Chest pain	1 (17)	0 (0)	0 (0)	0 (0)	1 (4)	0 (0)
Mucosal inflammation	0 (0)	0 (0)	1 (5)	0 (0)	1 (4)	0 (0)
Laryngitis	1 (17)	0 (0)	0 (0)	0 (0)	1 (4)	0 (0)
Groin pain	0 (0)	0 (0)	1 (5)	0 (0)	1 (4)	0 (0)
Headache	0 (0)	0 (0)	1 (5)	0 (0)	1 (4)	0 (0)
Exertional dyspnoea	0 (0)	0 (0)	1 (5)	0 (0)	1 (4)	0 (0)
Oropharyngeal pain	1 (17)	0 (0)	0 (0)	0 (0)	1 (4)	0 (0)
Pleuritic pain	1 (17)	0 (0)	0 (0)	0 (0)	1 (4)	0 (0)
Throat irritation	1 (17)	0 (0)	0 (0)	0 (0)	1 (4)	0 (0)
Rash	0 (0)	0 (0)	1 (5)	0 (0)	1 (4)	0 (0)
Macular rash	1 (17)	0 (0)	0 (0)	0 (0)	1 (4)	0 (0)
Decreased weight	1 (17)	0 (0)	0 (0)	0 (0)	1 (4)	0 (0)

a Treatment-related adverse events include all treatment-emergent adverse events that had a reasonable possibility of being related to trebananib or bevacizumab therapy; all patients received ≥1 dose of trebananib.

b Treatment-related adverse event was arterial haemorrhage in a patient with squamous cell carcinoma.

c Treatment-related adverse event was a DLT of tumour haemorrhage in a patient with squamous cell carcinoma.

Across the dose cohorts of trebananib plus motesanib (cohorts 2 and 4, *n =* 11), the most common treatment-related adverse events included hypertension, diarrhoea, nausea, fatigue, vomiting, and decreased appetite (Table 3). Two patients (18%) had grade ≥ 3 treatment-related adverse events; those events were all grade 3 and included hypertension (*n* = 1) in a patient with ovarian cancer, and leukoencephalopathy (*n* = 1) (the patient with leukoencephalopathy was not on antihypertensive medication previously) and intestinal perforation (*n* = 1) in a patient with ureteral cancer; the events of hypertension and leukoencephalopathy were not considered to be related to trebananib treatment. No grade 4 treatment-related adverse events occurred. Two patients died from renal failure (*n =* 1) and respiratory failure (*n =* 1), which was not considered to be treatment-related.

**Table 3:** Patient incidence of treatment–related adverse events in the trebananib plus motesanib cohorts

	Cohort 2		Cohort 4		Cohorts 2–4	
	Trebananib 3 mg kg^−1^ + motesanib 75 mg (*n* = 8)		Trebananib 3 mg kg^−1^ + motesanib 125 mg (*n* = 3)		Trebananib + motesanib (*n* = 11)	
Patients with any treatment-related adverse event^a^, *n* (%)	7 (88)		2 (67)		9 (82)	
Grade 3	2 (25)^b^		0 (0)		2 (18)	
Grade 4	0 (0)		0 (0)		0 (0)	
Grade 5	0 (0)		0 (0)		0 (0)	
**Treatment-related adverse events occurring in one or more treatment arms,** *n* **(%)**	**All grades**	**Grade ≥ 3**	**All grades**	**Grade ≥ 3**	**All grades**	**Grade ≥ 3**
Hypertension	3 (38)	1 (13)	1 (33)	0 (0)	4 (36)	1 (9)
Diarrhoea	3 (38)	0 (0)	1 (33)	0 (0)	4 (36)	0 (0)
Nausea	2 (25)	0 (0)	1 (33)	0 (0)	3 (27)	0 (0)
Fatigue	3 (38)	0 (0)	0 (0)	0 (0)	3 (27)	0 (0)
Vomiting	1 (13)	0 (0)	1 (33)	0 (0)	2 (18)	0 (0)
Decreased appetite	1 (13)	0 (0)	1 (33)	0 (0)	2 (18)	0 (0)
Hypothyroidism	1 (13)	0 (0)	0 (0)	0 (0)	1 (9)	0 (0)
Blurred vision	0 (0)	0 (0)	1 (33)	0 (0)	1 (9)	0 (0)
Abdominal pain	1 (13)	0 (0)	0 (0)	0 (0)	1 (9)	0 (0)
Lower abdominal pain	0 (0)	0 (0)	1 (33)	0 (0)	1 (9)	0 (0)
Cheilitis	1 (13)	0 (0)	0 (0)	0 (0)	1 (9)	0 (0)
Gastro-oesophageal reflux disease	1 (13)	0 (0)	0 (0)	0 (0)	1 (9)	0 (0)
Intestinal perforation^c^	1 (13)	1 (13)	0 (0)	0 (0)	1 (9)	1 (9)
Early satiety	1 (13)	0 (0)	0 (0)	0 (0)	1 (9)	0 (0)
Noncardiac chest pain	1 (13)	0 (0)	0 (0)	0 (0)	1 (9)	0 (0)
Peripheral oedema	1 (13)	0 (0)	0 (0)	0 (0)	1 (9)	0 (0)
Increased alanine aminotransferase	0 (0)	0 (0)	1 (33)	0 (0)	1 (9)	0 (0)
Hypomagnesaemia	0 (0)	0 (0)	1 (33)	0 (0)	1 (9)	0 (0)
Arthralgia	1 (13)	0 (0)	0 (0)	0 (0)	1 (9)	0 (0)
Myalgia	0 (0)	0 (0)	1 (33)	0 (0)	1 (9)	0 (0)
Tumour pain	1 (13)	0 (0)	0 (0)	0 (0)	1 (9)	0 (0)
Headache	0 (0)	0 (0)	1 (33)	0 (0)	1 (9)	0 (0)
Leukoencephalopathy	1 (13)	1 (13)	0 (0)	0 (0)	1 (9)	1 (9)
Urinary bladder haemorrhage	0 (0)	0 (0)	1 (33)	0 (0)	1 (9)	0 (0)
Sexual dysfunction	0 (0)	0 (0)	1 (33)	0 (0)	1 (9)	0 (0)
Rash	1 (13)	0 (0)	0 (0)	0 (0)	1 (9)	0 (0)

a Treatment-related adverse events include all treatment-emergent adverse events that had a reasonable possibility of being related to trebananib or motesanib therapy; all patients received ≥1 dose of trebananib.

b One patient had two grade 3 adverse events (intestinal perforation, leukoencephalopathy).

c Intestinal perforation for this patient was rated as a grade 3 treatment-related adverse event and classified as a DLT.

### Pharmacokinetics

**Figure 1:**
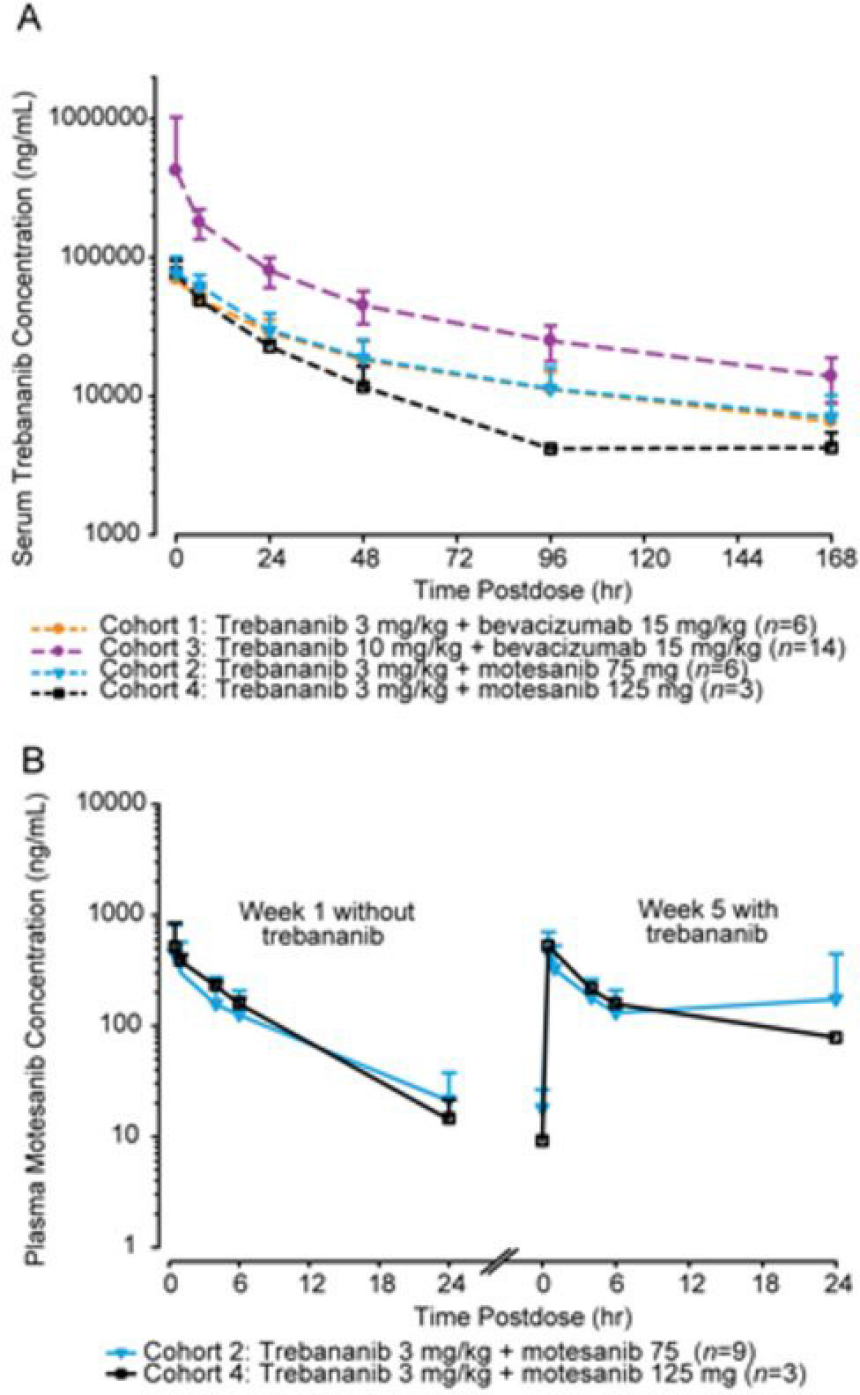
Pharmacokinetic concentration-time profiles **(A)** mean (+ SD) serum concentration-time profiles of trebananib at week 4 after 3 mg kg^−1^ IV QW infusions of trebananib in combination with motesanib and after 3 and 10 mg kg^−1^ IV QW infusions of trebananib with bevacizumab. **(B)** mean (+ SD) plasma concentration-time profiles of motesanib after 75 or 125 mg oral QD dosing of motesanib in combination with trebananib 3 mg kg^−1^.

The mean (+SD) serum concentration-time profiles of trebananib at steady state at week 4 following weekly IV infusions of trebananib in combination with bevacizumab or motesanib are shown in Figure 1A. Values for PK parameters of trebananib in this study are listed in Table 4 and are similar to those reported for trebananib monotherapy in a phase 1 study [16], consistent with a lack of effect of bevacizumab or motesanib on trebananib PK. Only limited data (*n =* 3) was available for cohort 4. The mean plasma concentration profiles of motesanib at 75 mg or 125 mg when coadministered with trebananib 3 mg kg^−1^ were similar to their profiles observed without the addition of trebananib (Table 4; Figure 1B).

**Table 4:** Summary of Trebananib Pharmacokinetic Parameters

	Trebananib 3 mg kg^−1^			Trebananib 10 mg kg^−1^	Motesanib 75 mg		Motesanib 125 mg	
**Descriptive statistics**	Bev 15 mg kg^−1^	Mot 75 mg	Mot 125 mg	Bev 15 mg kg^−1^	Without Trebananib	Trebananib 3 mg kg^−1^	Without Trebananib	Trebananib 3 mg kg^−1^
**T**_max_ **(h)**^a^								
N	6	6	3	14	9	5	3	2
**Mean**	0.55	0.57	0.53	0.50	1.1	1.0	1.0	1.0
**%CV**	6.1	160	12	11	100	200	37	NA
**C**_max_ **(μg ml^−1^)**^b^								
N	6	6	3	14	9	5	3	2
**Mean**	69.6	82.0	78.1	421	0.466	0.507	0.559	0.541
**%CV**	15.0	160	22	143	75.9	40.6	53.2	NA
**AUCtou (μg·h ml^−1^)**^c^								
N	6	6	3	14	9	5	3	2
**Mean**	2930	3230	2200	9040	2.58	3.18	3.13	3.92
**%CV**	24.5	26.5	21.0	32.0	68.1	89.7	12.2	NA
**CL (ml h^−1^ kg^−1^)**						**C24 (μg ml^−1^)**		
N	6	6	3	14	9	3	3	2
**Mean**	0.873	0.747	1.22	1.03	20.9	173	14.2	78.7
**%CV**	26.5	42.1	21.3	33.9	79.6	159	50.7	NA

a T_max_ = Time to reach C_max_; reported as median and range

b C_max_ = Maximum observed concentration during a dosing interval

c AcUC = Area under the concentration-time curve from time 0 to 168 hours for trebananib and time 0 to 24 hours for motesanib

AUCtou = Area under the concentration-time curve in a dosing interval at steady state;

Abbreviations: Bev = bevacizumab; CL = Clearance; C24 = concentration 24 hours after the start of infusion;

Mot = motesanib; NA = Not applicable; CV = coefficient of variation.

### Immunogenicity

Serum samples for anti-trebananib antibody analyses were available for all 36 patients. In both cohorts 1 and 3 (trebananib plus bevacizumab), one patient each developed binding antibodies during study treatment. Both patients continued to test positive for binding antibodies at the end of the study. One patient in cohort 2 was transiently positive for binding antibodies. No binding antibodies were detected in cohort 4. The baseline samples for one patient in cohort 1 and two patients in cohort 2 neutralised the trebananib activities in the neutralising activity assay, but did not demonstrate positivity in the immunoassay. Therefore, it was concluded that the samples did not have neutralising antibodies. No patient developed neutralising antibodies during the study period.

### Biomarkers

Changes in biomarker levels during treatment relative to baseline were statistically significant for two of the five tested analytes. There was a nominally significant increase in serum levels of PLGF in each cohort, except in cohort 4 (*P-*values ranged from 0.03 to 0.12 across timepoints; Supplemental Data, Figure 2A), which may be related to the limited number of available patient data (*n =* 3) for that cohort. Increases in serum levels of sVCAM-1 during treatment compared with baseline were statistically significant in each cohort (Supplemental Data, Figure 2B). Closer examination reveals a steplike pattern with an initial increase of sVCAM-1 levels on treatment day 4 followed by another elevation on day 9. In cohort 2, sVCAM-1 returned to pretreatment levels on day 50.

sKit was significantly (*P* < 0.01) elevated in cohort 3 on days 2 and 4. The remaining analytes remained largely unchanged over time for any of the treatment cohorts (data not shown). VEGF levels for either dose cohort of trebananib plus bevacizumab could not be detected as the result of assay interference from bevacizumab. There were no correlations between any of the biomarkers and tumour response (data not shown).

### Tumour response

**Figure 2:**
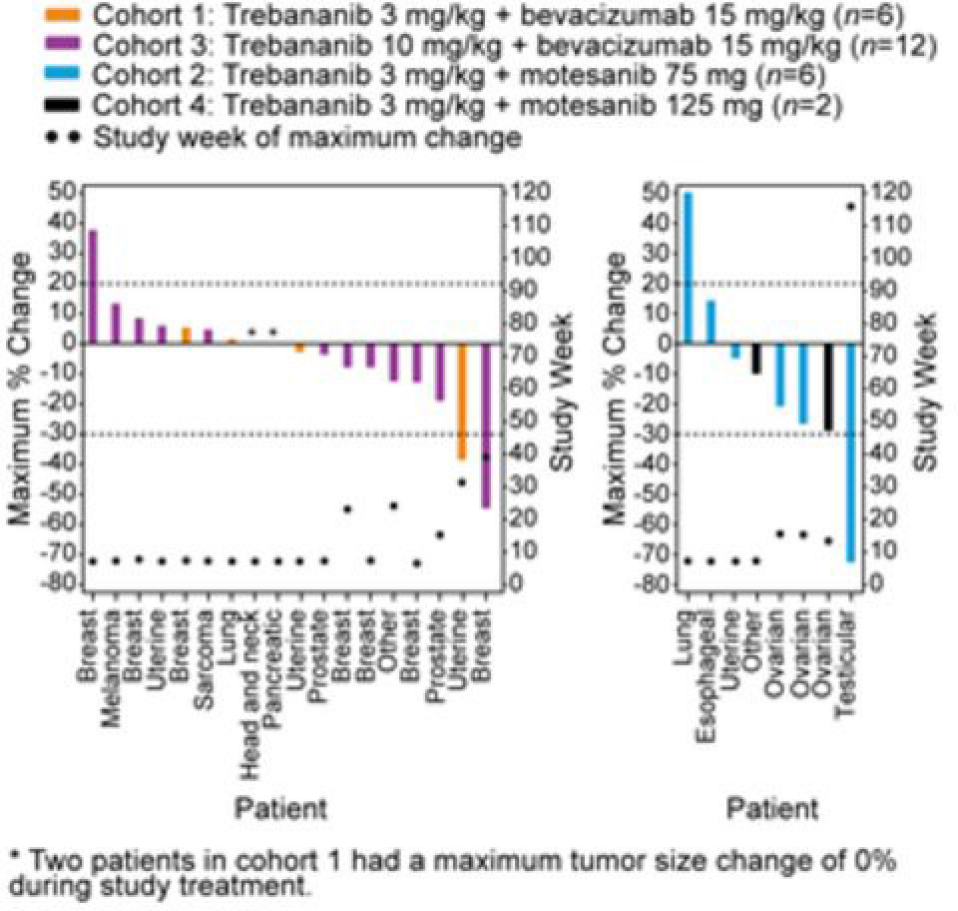
Tumour response by RECIST 1.0 Maximum percent change (measured by sum of longest diameters) in tumour burden by tumour type for each patient and study week when maximum percent change in tumour burden for each patient occurred.

All patients who received ≥1 dose of trebananib (*n* = 36) were initially considered for tumour response assessment. Tumour response by RECIST 1.0 [24] was available for 26 patients (Figure 2).

In the trebananib plus bevacizumab arms (cohorts 1 and 3), 25 patients received ≥1 dose of trebananib. Of these, 18 patients had response measurements by RECIST. Postbaseline tumour response assessments were not conducted for seven patients due to rapid clinical progression (*n* = 4), death (*n* = 1), and incorrect imaging (*n* = 2). Across cohorts 1 and 3, no patient achieved a complete response; two patients had a confirmed partial response. One of the patients with a partial response was in cohort 1 and had prostate cancer (duration of response, 16.3 weeks); the other patient was in cohort 3 and had breast cancer (duration of response, 68.1 weeks; this patient did not develop progressive disease by the data analysis cutoff date). The median percent change in tumour size was −1.23% across cohorts 1 and 3. Eleven patients had stable disease by RECIST criteria as their best response. Five patients with prostate (*n* = 2), thyroid (*n* = 1), breast (*n* = 1), and pancreatic cancer (*n* = 1) had stable disease >6 months (range: 28.6–84.1 weeks) while receiving study treatment.

In the trebananib plus motesanib arms (cohorts 2 and 4), 11 patients were evaluable. Eight patients had response measurements by RECIST. Postbaseline tumour responses could not be evaluated for three patients as the result of withdrawal of consent (*n* = 2) and clinical progression (*n* = 1). One patient with testicular cancer achieved a confirmed partial response that lasted 85.0 weeks (Supplemental Data, Figures 3A & B); the patient remained on the study for 116 weeks. The median percent change in tumour size was −15.06% across cohorts 2 and 4. Five patients with ovarian (*n* = 3), adrenal cortical (*n* = 1), and uterine cancer (*n* = 1) had stable disease by RECIST criteria, with the response of one patient with ovarian cancer lasting >6 months (41.0 weeks).

## DISCUSSION

The current study of trebananib administered intravenously QW in combination with bevacizumab or motesanib in patients with advanced cancer with considerable pre-treatment (69% of patients had received ≥3 lines of prior chemotherapy), shows that with both combinations, a true maximum tolerated dose was not achieved. Overall, the adverse events were similar to those toxicities described for trebananib, bevacizumab, and motesanib alone [16, 21, 22, 28]. Previous studies have demonstrated that the combination of two VEGF pathway inhibitors is generally not well tolerated in patients with cancer [29, 30]. In this study, it appeared that a less severe toxicity profile was associated with the combination of trebananib plus bevacizumab or motesanib when compared with toxicity profiles typically associated with treatments combining two VEGF pathway inhibitors.

One DLT occurred in cohort 3 when trebananib was combined with bevacizumab, and two deaths were thought to be at least possibly related to both trebananib and bevacizumab treatment per the clinical investigator's assessment. Those deaths occurred in head and neck cancer patients with squamous cell pathology and extensive disease to the lung and neck. One of those two patients developed severe neck pain and a new mass on the left neck 5 days after the first study drug dosing; 4 days later, the patient died with haemorrhage from the mouth. The second patient developed a massive haemorrhage from the mouth and nose and died after eight doses of trebananib and three doses of bevacizumab. In both instances, patients had tumours with squamous cell pathology, which at the start of the study was not an exclusion criterion for the treatment combination. Extensive experience with bevacizumab has shown that patients with squamous cell lung cancer are at high risk for tumour bleeding (31%) with fatal or serious bleeding events [31], which has led to a warning against using bevacizumab in squamous cell lung cancer [32]. Similarly, a study with bevacizumab and pemetrexed in recurrent or metastatic squamous cell head and neck cancer showed a high rate (15%) of grade 3 to 5 bleeding events [33]. It is unclear as to how much trebananib contributed to the deaths in the current study. The most common treatment-related adverse events seen with the trebananib plus bevacizumab combination included fatigue (32%), diarrhoea (16%), constipation (12%), nausea (12%), and epistaxis (12%). Of the 25 patients who received the combination, 12% had grade ≥ 3 treatment-related adverse events. There appeared to be no noticeable increase in hypertension, proteinuria, or intestinal perforation beyond what would be expected with bevacizumab alone.

Both trebananib [17] and bevacizumab [34] are likely active as single agents in combination with chemotherapy and, therefore, their combination has a potential risk of increasing the frequency or severity of toxicities that are typically observed during trebananib or bevacizumab monotherapy, such as peripheral oedema for trebananib [16] or intestinal perforation for bevacizumab [35]. All four patients with ovarian cancer who were enrolled in our study received trebananib plus motesanib and did not experience any signs of intestinal perforation, with three of the four patients having some level of minor response. Although we did not enrol any patients with ovarian cancer in the trebananib plus bevacizumab arms, the lack of intestinal perforation in the patients with ovarian cancer in the trebananib plus motesanib arms may suggest that combining trebananib and bevacizumab does not have a high incidence of intestinal perforation in a population with ovarian cancer. In addition, Karlan *et al* [17] showed recently that treatment with trebananib in combination with paclitaxel was not associated with a higher incidence of intestinal perforations in patients with recurrent ovarian cancer relative to a placebo group. This finding suggests that a combination of trebananib and bevacizumab may be feasible without significantly increasing the intestinal perforation rate over bevacizumab alone. A dose-response effect was also reported in the study by Karlan and colleagues, with improvement of PFS at trebananib 10 mg kg^−1^ (PFS, 2.6 months) versus trebananib 3 mg kg^−1^ (PFS, 1.1 months). In our study, we showed that trebananib plus bevacizumab was tolerable at doses up to 10 mg kg^−1^ for trebananib and 15 mg kg^−1^ for bevacizumab. Higher doses may be achievable with trebananib at 15 mg kg^−1^ and bevacizumab at 15 mg kg^−1^ given the lack of synergistic toxicities seen at the current tested dose levels.

The most common treatment-related adverse events associated with the trebananib and motesanib combination included hypertension (36%), diarrhoea (36%), nausea (27%), fatigue (27%), vomiting (18%), and decreased appetite (18%). These findings are similar to the adverse events seen with motesanib alone. The common toxicities previously associated with trebananib monotherapy, such as peripheral oedema, were not seen in this combination and may be reflective of the lower dose of trebananib at 3 mg kg^−1^ administered in the motesanib combination cohorts. The lack of haematologic adverse events that are typically observed with other combinations of VEGFR tyrosine kinase inhibitors and VEGFR axis antibodies, such as bevacizumab plus sorafenib [2] or bevacizumab plus sunitinib [36], was encouraging. Patients received trebananib a median of 9.5 and 11.0 weeks in cohorts 2 and 4, respectively; motesanib was administered a median of 67 and 87 days in those corresponding cohorts. One limitation of the design of this trial with a standard 3+3 design was the short DLT window (28 days), which did not adequately assess for long–term ongoing toxicities such as grade 2 nausea.

Although assessment of efficacy was not a primary objective of the study, the combinations showed some interesting clinical activity. In cohort 3, a prostate cancer patient with significant lung and bone metastases had a partial response despite an increase in this patient's prostate–specific antigen (data not shown). A second patient with breast cancer in cohort 3 had a partial response lasting 68 weeks. In addition, a patient with testicular cancer in cohort 2 showed a partial response lasting 85 weeks. Finally, six patients had stable disease lasting >6 months while on study; they were two patients with prostate cancer, and one patient each with ovarian, thyroid, breast, or pancreatic cancer.

The PK of motesanib did not appear to be affected when administered in combination with trebananib. In addition, trebananib concentration-time profiles in this study were comparable to those of the monotherapy trial [16], suggesting that trebananib PK was not affected by the coadministration of bevacizumab or motesanib. The presence of the binding anti-drug antibodies did not appear to affect the PK of trebananib.

We explored five potential biomarkers of angiogenesis to measure the biological activity of the trebananib combinations. Only serum PLGF and sVCAM-1 showed significant increases in each cohort. In a combination study of trebananib with chemotherapy, a rise in PLGF and sVCAM-1 concentrations in each cohort (trebananib plus FOLFOX-4, carboplatin/paclitaxel, or docetaxel) was also observed [37]. Other antiangiogenic agents, such as sunitinib [38, 39], bevacizumab [40], and motesanib [21], have shown increases in PLGF, which may reflect a compensatory mechanism (ie, increase in upstream proangiogenic signalling) when blocking downstream pathways. Based on our data, there is not a significant difference in changes in PLGF and sVCAM-1 between the high and low dose levels of trebananib, except during week 50 for sVCAM-1. However, the number of samples in our study was small, and a larger cohort is needed to come to a definitive conclusion whether trebananib dose levels affect PLGF or sVCAM-1 changes during treatment.

In conclusion, the combination of VEGF pathway inhibitors in the treatment of cancer has been previously associated with high toxicity. In this study, results suggest that the combinations of trebananib, an angiopoietin pathway inhibitor, with the anti-VEGF agents bevacizumab or motesanib in patients with advanced solid tumours may be associated with a less severe toxicity profile when compared with combination treatments targeting only the VEGF pathway. Furthermore, the combinations tested in the present study did not appear to exacerbate toxicities that are typically observed when these treatments are administered as monotherapies. Although the trial did not show clear evidence of synergistic activity, the treatment combinations were associated with antitumour activity across a broad range of tumour types. The data support larger studies of trebananib in combination with VEGF pathway inhibitors. Various phase 2 clinical trials have been conducted, including studies of trebananib with sorafenib in patients with advanced hepatocellular carcinoma (ClinicalTrials.gov NCT00872014), trebananib with sunitinib [41] or sorafenib [42] in patients with renal cell cancer, and trebananib with bevacizumab in patients with breast cancer (ClinicalTrials.gov NCT00511459).

## MATERIALS AND METHODS

### Patients

Key eligibility criteria included age ≥18 years; a diagnosis of advanced solid tumors refractory to standard treatment; measurable or evaluable (nonmeasurable) disease per Response Evaluation Criteria in Solid Tumors (RECIST) guidelines (v.1.0) [24]; an Eastern Cooperative Oncology Group (ECOG) performance status ≤2; and adequate hematological, renal, and hepatic function. Excluded were patients with a prior bleeding diathesis, gastrointestinal surgery or disease, thrombosis or pulmonary embolism or cardiovascular events during the past year; a diagnosis of ovarian cancer while receiving bevacizumab; a diagnosis of lung cancer with tumor lesions ≥3 cm; or chronic uncontrolled hypertension, chronic hepatitis, or symptomatic or untreated central nervous system metastases; a treatment history with angiopoietin or VEGF inhibitors or radiation therapy to the abdomen; recent treatment with anticancer or palliative radiation; or current treatment regimens containing strong CYP 3A inhibitors, immune modulators, St. John's wort, or coumarin anticoagulants ≥2 mg/day. Because two patients with squamous cell head and neck cancer developed grade 5 hemorrhage, the protocol was amended to exclude patients with head and neck cancer or lung squamous cell tumors. All patients provided written informed consent. An institutional review board of each study center approved all study procedures.

### Study design and treatment

This open-label, dose escalation phase 1b study was carried out at five centres in the United States and examined trebananib in combination with bevacizumab, motesanib, sorafenib, or sunitinib (Supplemental Data, Figure 4). Patients in the cohorts receiving trebananib combined with bevacizumab or motesanib had diagnoses of advanced solid tumours across various tumour types. In contrast, patients in the cohorts receiving trebananib combined with sorafenib or sunitinib had primary diagnoses of renal cell cancer. To avoid invalid comparisons between those two patient groups, only findings from cohorts receiving trebananib combined with bevacizumab or motesanib are presented in the current report. Those cohorts were cohort 1 (trebananib 3 mg kg^−1^ + bevacizumab 15 mg kg^−1^), cohort 2 (trebananib 3 mg kg^−1^ + motesanib 75 mg), cohort 3 (trebananib 10 mg kg^−1^ + bevacizumab 15 mg kg^−1^), and cohort 4 (trebananib 3 mg kg^−1^ + motesanib 125 mg). Trebananib and bevacizumab were administered intravenously QW and once every 3 weeks (Q3W), respectively; motesanib was self-administered orally by patients once daily (QD). For each cohort, trebananib was initiated on day 1 of the second week; the first dose of bevacizumab or motesanib was administered on day 1 of the first week. For patients in cohorts 1 and 3, trebananib was administered at least 30 minutes following bevacizumab infusion on days when both therapies were administered; for patients in cohorts 2 and 4, trebananib and motesanib had to be administered simultaneously. Trebananib dose levels were selected based on the first-in-human monotherapy study showing trebananib up to 30 mg kg^−1^ to be tolerable [16]. The study protocol was amended to remove trebananib 30 mg/kg, consistent with dosing in a planned phase 2 study. Tolerable dose levels of bevacizumab and motesanib were established upon review of completed clinical trials [21, 25].

An initial three patients entered cohort 1 (trebananib plus bevacizumab) and cohort 2 (trebananib plus motesanib). If none of the initial three patients experienced a dose-limiting toxicity (DLT) in cohort 1 or 2, up to six patients were enrolled to receive a higher dose (10 mg kg^−1^) of trebananib with the same dose (15 mg kg^−1^) of bevacizumab in cohort 3 and the same dose (3 mg kg^−1^) of trebananib with a higher dose (125 mg) of motesanib in cohort 4. If one of the initial three patients in cohort 1 or 2 experienced a DLT, that cohort was expanded to six patients. In this expanded cohort 1 or 2, if fewer than three patients experienced a DLT, up to six patients were enrolled into the corresponding higher dose cohort 3 or 4. If two or more of the initial three patients in cohort 1 or 2 experienced a DLT, no additional patients were enrolled into the respective higher dose cohorts. A maximum of 10 additional patients could be added to one cohort of each treatment combination (trebananib plus bevacizumab and trebananib plus motesanib), irrespective of dosage. Since patients received only bevacizumab or motesanib for the first study week before trebananib dosing was initiated, any patient who experienced a DLT during the first study week was withdrawn and replaced with a new patient. Patients who discontinued the study during the first study month were also withdrawn and replaced. DLTs occurring during the first study week did not influence the dose escalation process. For any patient in a given cohort who experienced a treatment deviation with trebananib during the first study month, an additional patient was enrolled.

A DLT was any related, grade 3 or higher haematologic or nonhaematologic toxicity during the initial 28 study days, except for the following adjustments (which were required for those AEs to be considered dose limiting): transient grade 3 infusion reactions lasting more than 2 hours; grade 3 fatigue for more than 7 days; grade 3 or 4 nausea, diarrhoea, or vomiting despite maximum supportive care; grade 3 or 4 neutropenia with fever above 38.5°C; grade 4 neutropenia for more than 7 days; grade 4 thrombocytopenia, anaemia, and hypertension; grade 4 aspartate or alanine aminotransferase greater than 10 times the upper limit of normal.

Primary endpoints were the incidence of adverse events and PK of trebananib and motesanib. Secondary endpoints were anti–trebananib antibody formation, the biomarker profile, and tumor response.

Blood pressure monitoring. Patients' blood pressure was measured based on the Seventh Report of the Joint National Committee on Prevention, Detection, Evaluation, and Treatment of High Blood Pressure (JNC 7). All measurements were conducted on site and according to the following guidelines: caffeine, exercise, and smoking were avoided for at least 30 minutes before the measurement; patients should be seated for at least 5 minutes in a chair (rather than on an examination table), with feet on the floor, and arm supported at the heart level; an appropriately sized cuff (cuff bladder encircling at least 80% of the arm) was used to ensure accuracy. Blood pressure was measured according to the following schedule: screening, weeks 1 and 5 (predose, 1, 4, and 24 hours postdose); weeks 2, 3, 4, 6, and beyond (predose); end of study visit.

### Adverse events

Adverse events were recorded and classified following the Medical Dictionary for Regulatory Activities and subsequently graded according to the National Cancer Institute Common Terminology Criteria for Adverse Events (NCI-CTCAE v.3.0). Adverse events were determined by clinical evaluations and laboratory assessments at screening and on the first day of every study week. Unless otherwise noted, this report presents treatment-related adverse events as assessed by the clinical investigator based on a reasonable possibility that the event could be related to any of the study treatments. All patients who received ≥1 dose of any study agent were included in this analysis set.

### Pharmacokinetics

Motesanib PK was assessed by comparing week 1 versus week 4 or 5 (motesanib without and with trebananib, respectively). Serum samples for the evaluation of minimum and maximum observed trebananib concentrations in the plasma were collected immediately before dosing on day 1 of weeks 2, 3, 4, 5, 6, 8, and every 8 weeks thereafter until 4 weeks after the last dose of study treatment. In addition, intensive sampling was conducted at the end of infusion, and 6, 24, 48, and 96 hours following dosing on day 1 of weeks 4 or 5. The samples were analysed for trebananib concentrations employing a validated enzyme-linked immunosorbent assay [16].

Assessment of bevacizumab PK interaction with trebananib was not feasible due to bevacizumab accumulation between weeks 1 and 5. For motesanib, postdose samples were taken at 0.5, 1, 4, 6, and 24 hours during weeks 1 and 5 (without and with trebananib coadministration, respectively). Motesanib plasma concentrations were assessed with a validated liquid chromatography/mass spectrometry (LC-MS/MS) method at Cedra Corporation (Austin, TX).

### Immunogenicity

Serum samples were evaluated for immunogenicity of trebananib prior to dosing during weeks 1, 3, 5, 8, and every 4 weeks thereafter until study termination. Two validated assays, an electrochemiluminescence (ECL) immunoassay and ECL receptor-binding neutralising assay, were used to detect anti-trebananib binding and neutralizing antibodies, respectively [26]. A sample was defined as positive for neutralizing antibodies when it was positive for binding antibodies in the immunoassay and positive in the neutralizing activity assay.

### Biomarkers

Pharmacodynamic profiles of biomarkers were evaluated in serum samples obtained immediately before and 24 and 72 hours after dosing during weeks 1 and 2 and before dosing during weeks 3, 4, and 8 and every 8 weeks thereafter until the last study visit. Specific biomarkers assessed included angiogenic cytokines (VEGF, placental growth factor [PLGF]), soluble VEGFR-2, soluble c-Kit receptor (sKit), and soluble vascular cell adhesion molecule-1 (sVCAM-1). To quantify PLGF and VEGF levels, a three-plex sandwich immunoassay with electrochemiluminescent detection (Meso-Scale Discoveries [MSD], Gaithersburg, MD]) was used. VEGFR-2 and sKit levels were measured with a two-plex MSD assay. sVCAM-1 levels were quantified using specific ELISA kits (R&D Systems, Minneapolis, MN). All biomarker measurements were conducted as previously described [27]. The detection of VEGF was completely inhibited when bevacizumab was added to samples during assay validation. Therefore, VEGF results are not reported for cohorts 1 and 3.

### Tumour response evaluations

Computerized tomography (CT) or magnetic resonance imaging (MRI) assessment of measureable disease was conducted within a 4–week time window before treatment initiation and extended to 8 weeks for brain imaging. Tumour response was evaluated with CT or MRI at week 8 and every 8 weeks thereafter and categorized using RECIST, version 1.0 [24]. Patients who discontinued the study before week 8 were evaluated for tumour response during the last visit. All patients who received ≥1 dose of trebananib and had a baseline and at least one postdose assessment of tumour burden were included in this analysis set. Tumour burden was defined as the sum of the longest diameters (SLD) for up to 10 target lesions.

### Statistical analysis

Tolerability, PK, and tumor measurements and response results are expressed with descriptive statistics. For biomarker analyses, log–transformed analyte values were tested for changes from baseline to the treatment time points. Statistical significance (*P*<0.01) for change from baseline was based on the analysis of variance using an F–test.

## SUPPLEMENTARY FIGURES



## References

[1] Folkman J (2007). Angiogenesis: an organizing principle for drug discovery?. Nat Rev Drug Discov.

[2] Azad NS, Posadas EM, Kwitkowski VE, Steinberg SM, Jain L, Annunziata CM, Minasian L, Sarosy G, Kotz HL, Premkumar A, Cao L, McNally D, Chow C, Chen HX, Wright JJ, Figg WD (2008). Combination targeted therapy with sorafenib and bevacizumab results in enhanced toxicity and antitumor activity. J Clin Oncol.

[3] Jain RK (2005). Normalization of tumor vasculature: An emerging concept in antiangiogenic therapy. Science.

[4] Casanovas O, Hicklin DJ, Bergers G, Hanahan D (2005). Drug resistance by evasion of antiangiogenic targeting of VEGF signaling in late-stage pancreatic islet tumors. Cancer Cell.

[5] Burger RA (2011). Overview of anti-angiogenic agents in development for ovarian cancer. Gynecol Oncol.

[6] Hashizume H, Falcon BL, Kuroda T, Baluk P, Coxon A, Yu D, Bready JV, Oliner JD, McDonald DM (2010). Complementary actions of inhibitors of angiopoietin-2 and VEGF on tumor angiogenesis and growth. Cancer Res.

[7] Maisonpierre PC, Suri C, Jones PF, Bartunkova S, Wiegand SJ, Radziejewski C, Compton D, McClain J, Aldrich TH, Papadopoulos N, Daly TJ, Davis S, Sato TN, Yancopoulos GD (1997). Angiopoietin-2, a natural antagonist for Tie2 that disrupts in vivo angiogenesis. Science.

[8] Suri C, Jones PF, Patan S, Bartunkova S, Maisonpierre PC, Davis S, Sato TN, Yancopoulos GD (1996). Requisite role of angiopoietin-1, a ligand for the TIE2 receptor, during embryonic angiogenesis. Cell.

[9] Eklund L, Olsen BR (2006). Tie receptors and their angiopoietin ligands are context-dependent regulators of vascular remodeling. Exp Cell Res.

[10] Yancopoulos GD, Davis S, Gale NW, Rudge JS, Wiegand SJ, Holash J (2000). Vascular-specific growth factors and blood vessel formation. Nature.

[11] Oliner J, Min H, Leal J, Yu D, Rao S, You E, Tang X, Kim H, Meyer S, Han SJ, Hawkins N, Rosenfeld R, Davy E, Graham K, Jacobsen F, Stevenson S (2004). Suppression of angiogenesis and tumor growth by selective inhibition of angiopoietin-2. Cancer Cell.

[12] Bunone G, Vigneri P, Mariani L, Buto S, Collini P, Pilotti S, Pierotti MA, Bongarzone I (1999). Expression of angiogenesis stimulators and inhibitors in human thyroid tumors and correlation with clinical pathological features. Am J Pathol.

[13] Etoh T, Inoue H, Tanaka S, Barnard GF, Kitano S, Mori M (2001). Angiopoietin-2 is related to tumor angiogenesis in gastric carcinoma. Cancer Res.

[14] Sallinen H, Heikura T, Laidinen S, Kosma VM, Heinonen S, Yla-Herttuala S, Anttila M (2010). Preoperative angiopoietin-2 serum levels: a marker of malignant potential in ovarian neoplasms and poor prognosis in epithelial ovarian cancer. Int J Gynecol Cancer.

[15] Coxon A, Bready J, Min H, Kaufman S, Leal J, Yu D, Lee TA, Sun JR, Estrada J, Bolon B, McCabe J, Wang L, Rex K, Caenepeel S, Hughes P, Cordover D (2010). Context-dependent role of angiopoietin-1 inhibition in the suppression of angiogenesis and tumor growth: implications for AMG 386, an angiopoietin-1/2-neutralizing peptibody. Mol Cancer Ther.

[16] Herbst RS, Hong D, Chap L, Kurzrock R, Jackson E, Silverman JM, Rasmussen E, Sun YN, Zhong D, Hwang YC, Evelhoch JL, Oliner JD, Le N, Rosen LS (2009). Safety, pharmacokinetics, and antitumor activity of AMG 386, a selective angiopoietin inhibitor, in adult patients with advanced solid tumors. J Clin Oncol.

[17] Karlan BY, Oza AM, Richardson GE, Provencher DM, Hansen VL, Buck M, Chambers SK, Ghatage P, Pippitt CH, Brown JV, Covens A, Nagarkar RV, Davy M, Leath CA, Nguyen H, Stepan DE (2012). Randomized, double-blind, placebo-controlled phase II study of AMG 386 combined with weekly paclitaxel in patients with recurrent ovarian cancer. J Clin Oncol.

[18] Sandler A, Gray R, Perry MC, Brahmer J, Schiller JH, Dowlati A, Lilenbaum R, Johnson DH (2006). Paclitaxel-carboplatin alone or with bevacizumab for non-small-cell lung cancer. N Engl J Med.

[19] Hurwitz H, Fehrenbacher L, Novotny W, Cartwright T, Hainsworth J, Heim W, Berlin J, Baron A, Griffing S, Holmgren E, Ferrara N, Fyfe G, Rogers B, Ross R, Kabbinavar F (2004). Bevacizumab plus irinotecan, fluorouracil, and leucovorin for metastatic colorectal cancer. N Engl J Med.

[20] Polverino A, Coxon A, Starnes C, Diaz Z, DeMelfi T, Wang L, Bready J, Estrada J, Cattley R, Kaufman S, Chen D, Gan Y, Kumar G, Meyer J, Neervannan S, Alva G (2006). AMG 706, an oral, multikinase inhibitor that selectively targets vascular endothelial growth factor, platelet-derived growth factor, and kit receptors, potently inhibits angiogenesis and induces regression in tumor xenografts. Cancer Res.

[21] Rosen LS, Kurzrock R, Mulay M, Van Vugt A, Purdom M, Ng C, Silverman J, Koutsoukos A, Sun YN, Bass MB, Xu RY, Polverino A, Wiezorek JS, Chang DD, Benjamin R, Herbst RS (2007). Safety, pharmacokinetics, and efficacy of AMG 706, an oral multikinase inhibitor, in patients with advanced solid tumors. J Clin Oncol.

[22] Sherman SI, Wirth LJ, Droz JP, Hofmann M, Bastholt L, Martins RG, Licitra L, Eschenberg MJ, Sun YN, Juan T, Stepan DE, Schlumberger MJ (2008). Motesanib diphosphate in progressive differentiated thyroid cancer. N Engl J Med.

[23] Blumenschein GR, Kabbinavar F, Menon H, Mok TS, Stephenson J, Beck JT, Lakshmaiah K, Reckamp K, Hei YJ, Kracht K, Sun YN, Sikorski R, Schwartzberg L (2011). A phase II, multicenter, open-label randomized study of motesanib or bevacizumab in combination with paclitaxel and carboplatin for advanced nonsquamous non-small-cell lung cancer. Ann Oncol.

[24] Therasse P, Arbuck SG, Eisenhauer EA, Wanders J, Kaplan RS, Rubinstein L, Verweij J, Van Glabbeke M, van Oosterom AT, Christian MC, Gwyther SG (2000). New guidelines to evaluate the response to treatment in solid tumors. European Organization for Research and Treatment of Cancer, National Cancer Institute of the United States, National Cancer Institute of Canada. J Natl Cancer Inst.

[25] Hainsworth JD, Fang L, Huang JE, Karlin D, Russell K, Faoro L, Azzoli C (2011). BRIDGE: an open-label phase II trial evaluating the safety of bevacizumab + carboplatin/paclitaxel as first-line treatment for patients with advanced, previously untreated, squamous non-small cell lung cancer. J Thorac Oncol.

[26] Zhong ZD, Dinnogen S, Hokom M, Ray C, Weinreich D, Swanson SJ, Chirmule N (2010). Identification and inhibition of drug target interference in immunogenicity assays. J Immunol Methods.

[27] Bass MB, Sherman SI, Schlumberger MJ, Davis MT, Kivman L, Khoo HM, Notari KH, Peach M, Hei YJ, Patterson SD (2010). Biomarkers as predictors of response to treatment with motesanib in patients with progressive advanced thyroid cancer. J Clin Endocrinol Metab.

[28] Yang JC, Haworth L, Sherry RM, Hwu P, Schwartzentruber DJ, Topalian SL, Steinberg SM, Chen HX, Rosenberg SA (2003). A randomized trial of bevacizumab, an anti-vascular endothelial growth factor antibody, for metastatic renal cancer. N Engl J Med.

[29] Eskens FA, Verweij J (2006). The clinical toxicity profile of vascular endothelial growth factor (VEGF) and vascular endothelial growth factor receptor (VEGFR) targeting angiogenesis inhibitors; a review. Eur J Cancer.

[30] Stone RL, Sood AK, Coleman RL (2010). Collateral damage: toxic effects of targeted antiangiogenic therapies in ovarian cancer. Lancet Oncol.

[31] Johnson DH, Fehrenbacher L, Novotny WF, Herbst RS, Nemunaitis JJ, Jablons DM, Langer CJ, DeVore RF, Gaudreault J, Damico LA, Holmgren E, Kabbinavar F (2004). Randomized phase II trial comparing bevacizumab plus carboplatin and paclitaxel with carboplatin and paclitaxel alone in previously untreated locally advanced or metastatic non-small-cell lung cancer. J Clin Oncol.

[32] Genentech (2011). Avastin (bevacizumab): highlights of prescribing information.

[33] Argiris A, Karamouzis MV, Gooding WE, Branstetter BF, Zhong S, Raez LE, Savvides P, Romkes M (2011). Phase II trial of pemetrexed and bevacizumab in patients with recurrent or metastatic head and neck cancer. J Clin Oncol.

[34] Perren TJ, Swart AM, Pfisterer J, Ledermann JA, Pujade-Lauraine E, Kristensen G, Carey MS, Beale P, Cervantes A, Kurzeder C, du Bois A, Sehouli J, Kimmig R, Stahle A, Collinson F, Essapen S (2011). A phase 3 trial of bevacizumab in ovarian cancer. N Engl J Med.

[35] Hapani S, Chu D, Wu S (2009). Risk of gastrointestinal perforation in patients with cancer treated with bevacizumab: a meta-analysis. Lancet Oncol.

[36] Feldman DR, Baum MS, Ginsberg MS, Hassoun H, Flombaum CD, Velasco S, Fischer P, Ronnen E, Ishill N, Patil S, Motzer RJ (2009). Phase I trial of bevacizumab plus escalated doses of sunitinib in patients with metastatic renal cell carcinoma. J Clin Oncol.

[37] Mita AC, Takimoto CH, Mita M, Tolcher A, Sankhala K, Sarantopoulos J, Valdivieso M, Wood L, Rasmussen E, Sun YN, Zhong ZD, Bass MB, Le N, LoRusso P (2010). Phase 1 study of AMG 386, a selective angiopoietin 1/2-neutralizing peptibody, in combination with chemotherapy in adults with advanced solid tumors. Clin Cancer Res.

[38] Rini BI, Michaelson MD, Rosenberg JE, Bukowski RM, Sosman JA, Stadler WM, Hutson TE, Margolin K, Harmon CS, DePrimo SE, Kim ST, Chen I, George DJ (2008). Antitumor activity and biomarker analysis of sunitinib in patients with bevacizumab-refractory metastatic renal cell carcinoma. J Clin Oncol.

[39] Deprimo SE, Bello CL, Smeraglia J, Baum CM, Spinella D, Rini BI, Michaelson MD, Motzer RJ (2007). Circulating protein biomarkers of pharmacodynamic activity of sunitinib in patients with metastatic renal cell carcinoma: modulation of VEGF and VEGF-related proteins. J Transl Med.

[40] Willett CG, Boucher Y, Duda DG, di Tomaso E, Munn LL, Tong RT, Kozin SV, Petit L, Jain RK, Chung DC, Sahani DV, Kalva SP, Cohen KS, Scadden DT, Fischman AJ, Clark JW (2005). Surrogate markers for antiangiogenic therapy and dose-limiting toxicities for bevacizumab with radiation and chemotherapy: continued experience of a phase I trial in rectal cancer patients. J Clin Oncol.

[41] Atkins MB, Ravaud A, Gravis G, Drosik K, Demkow T, Tomczak P, Kracht K, Puhlmann M, Weinreich DM (2012). Safety and efficacy of AMG 386 in combination with sunitinib in patients with metastatic renal cell carcinoma (mRCC) in an open-label multicenter phase II study. ASCO Meeting Abstracts.

[42] Rini B, Szczylik C, Tannir NM, Koralewski P, Tomczak P, Deptala A, Dirix LY, Fishman M, Ramlau R, Ravaud A, Rogowski W, Kracht K, Sun YN, Bass MB, Puhlmann M, Escudier B (2012). AMG 386 in combination with sorafenib in patients with metastatic clear cell carcinoma of the kidney: A randomized, double-blind, placebo-controlled, phase 2 study. Cancer.

