# Supplementary material for: A phase 1b, open-label study of trebananib plus bevacizumab or motesanib in patients with solid tumours
Source: Oncotarget. 2014 Oct 23;5(22):11154–67. doi: 10.18632/oncotarget.2568 (PMC4294348; doi:10.18632/oncotarget.2568)
Supplement: Supplementary file 1 [file oncotarget-05-11154-s001.pdf]

## SUPPLEMENTARY FIGURES

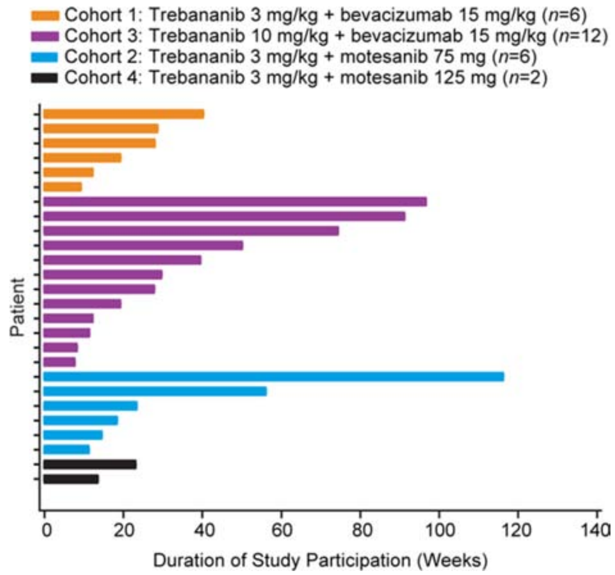


**Supplementary Figure S1: Number of weeks from enrollment to treatment termination across treatment cohorts for individual patients who had available tumour response measurements.**

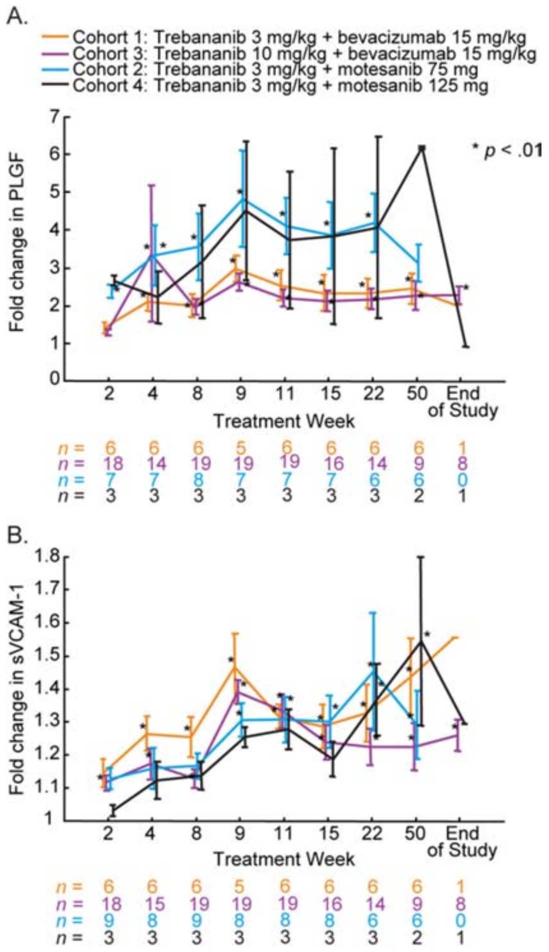


**Supplementary Figure S2: Fold change from baseline in concentrations of placental growth factor (PLGF) and soluble vascular cell adhesion molecule-1 (sVCAM-1) for all treatment cohorts during the course of the study.** Mean (± SE) fold change in PLGF **(A)** and sVCAM-1 **(B)**.

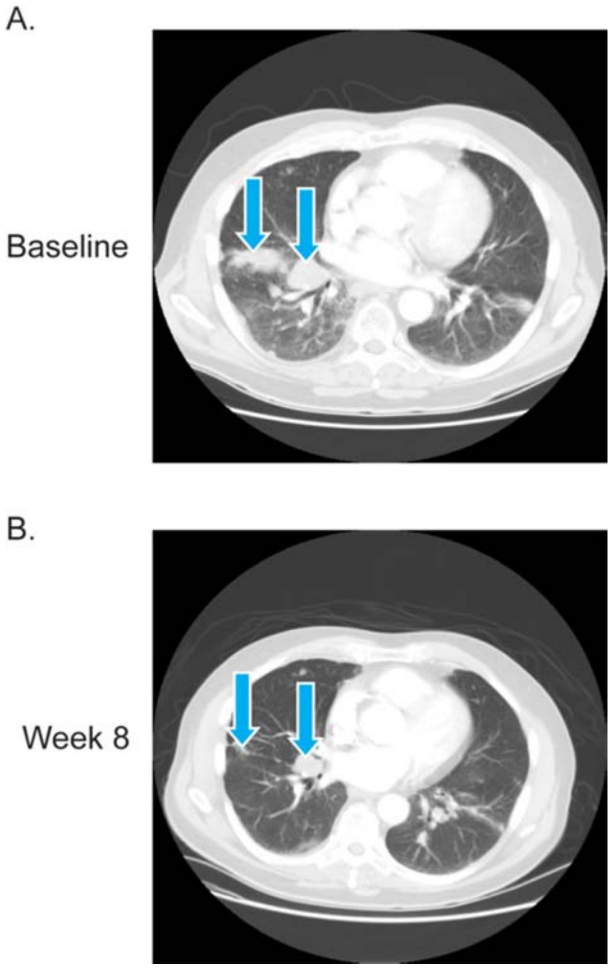


**Supplementary Figure S3: Computed tomography (CT) scan of two tumour lesions in the right middle lobe of the lung in a patient with testicular cancer at baseline (A) and after 8 weeks of treatment (B) with trebananib 3 mg $kg^{-1}$ IV QW plus motesanib 75 mg oral QD.** Arrows denote tumour lesions.

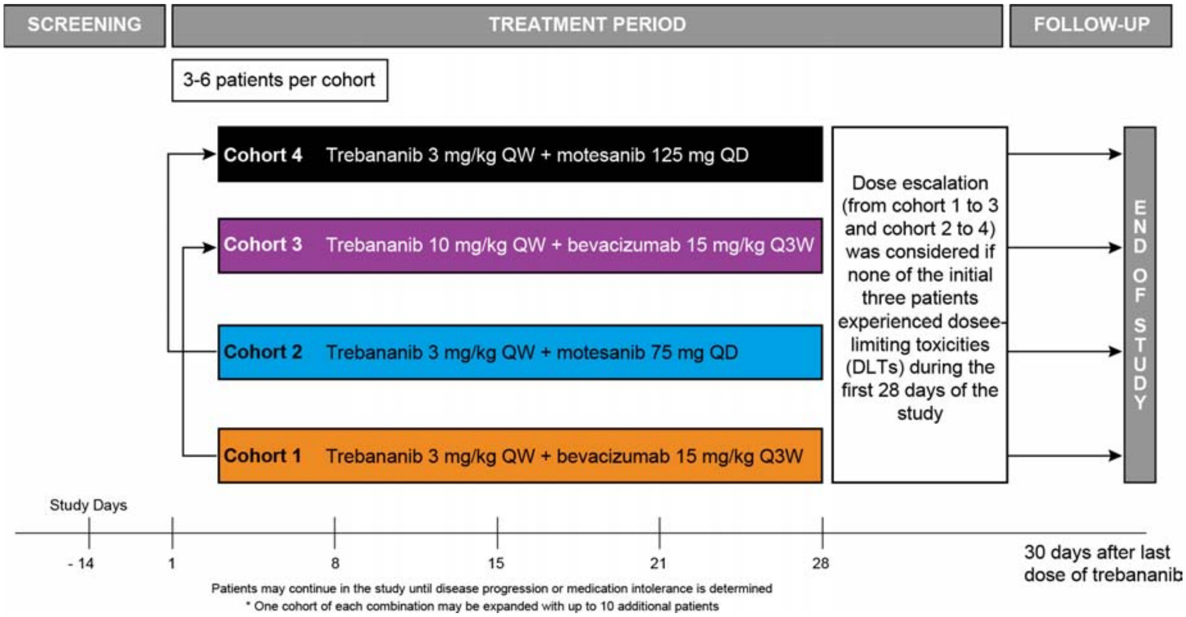


**Supplementary Figure S4: Study design.**
